# Measurement of Subvisible Particulates in Lyophilised *Erwinia chrysanthemi*l-asparaginase and Relationship with Clinical Experience

**DOI:** 10.1208/s12248-014-9612-9

**Published:** 2014-05-23

**Authors:** David Gervais, Tim Corn, Andrew Downer, Stuart Smith, Alan Jennings

**Affiliations:** 1 Microbiology Services, Development and Production, Public Health England, Porton Down, Wiltshire, Salisbury, SP4 0JG UK; 2 EUSA Pharma, Magdalen Centre, Oxford Science Park, Oxon, OX4 4GA UK

**Keywords:** *Erwinia*, flow-imaging microscopy, l-asparaginase, light obscuration, subvisible particulates

## Abstract

In order to generate further characterisation data for the lyophilised product *Erwinia chrysanthemi* l-asparaginase, reconstituted drug product (DP; marketed as Erwinase or Erwinaze) was analysed for subvisible (2–10 μm) particulate content using both the light obscuration (LO) method and the newer flow-imaging microscopy (FIM) technique. No correlation of subvisible particulate counts exists between FIM and LO nor do the counts correlate with activity at both release and on stability. The subvisible particulate content of lyophilised Erwinia l-asparaginase appears to be consistent and stable over time and in line with other parenteral biopharmaceutical products. The majority (ca. 75%) of subvisible particulates in l-asparaginase DP were at the low end of the measurement range by FIM (2–4 μm). In this size range, FIM was unable to definitively classify the particulates as either protein or non-protein. More sensitive measurement techniques would be needed to classify the particulates in lyophilised l-asparaginase into type (protein and non-protein), so the LO technique has been chosen for on-going DP analyses. *E. chrysanthemi* l-asparaginase has a lower rate of hypersensitivity compared with native *Escherichia coli* preparations, but a subset of patients develop hypersensitivity to the *Erwinia* enzyme. A DP lot that had subvisible particulate counts on the upper end of the measurement range by both LO and FIM had the same incidence of allergic hypersensitivity in clinical experience as lots at all levels of observed subvisible particulate content, suggesting that the presence of l-asparaginase subvisible particulates is not important with respect to allergic response.

## INTRODUCTION

l-asparaginase or l-asparagine amidohydrolase (EC 3.5.1.1) is an enzyme that catalyses the conversion of l-asparagine (l-Asn) to l-aspartic acid (l-Asp), with the evolution of ammonia. The enzyme is routinely used in chemotherapy regimens for the treatment of acute lymphoblastic leukaemia (ALL) (1). In this chemotherapeutic setting, the enzyme is used to decrease the serological concentration of l-Asn, thus depriving leukaemic cells of an essential amino acid nutrient (2). Clinical preparations of the enzyme are derived from two bacteria: *Escherichia coli* l-asparaginase (EcA) and *Erwinia chrysanthemi* l-asparaginase (ErA). In most instances, ErA is used in patients who develop hypersensitivity to EcA (3,4). As for all parenteral products, the measurement of subvisible particulates (SbVP) is an important consideration for manufacture and clinical supply of both ErA and EcA. ErA drug product (DP) is supplied as lyophilized protein for administration after reconstitution.

The formation of protein aggregates in a biopharmaceutical product must be controlled and understood and is an important process to consider when designing DP formulations. Protein aggregates may take the form of usually smaller, soluble aggregates or larger, insoluble aggregates including SbVP (5). Lyophilisation, as a process which removes water from the protein matrix and therefore brings protein molecules into closer proximity, must be regarded as a potential aggregation-inducing step (6), requiring further analysis with respect to SbVP. Multi-subunit proteins, such as the tetrameric 140 kDa (35 kDa subunit) l-asparaginase, may also face issues during lyophilisation such as loss of quaternary structure and possible loss of activity (7,8). The degree to which aggregation may occur in reconstituted lyophilized DP formulations varies from protein to protein, and the degree of aggregation may be reduced or lowered by additions of excipients such as sucrose or trehalose (9,10).

Concern with regard to the potential for undesired immunogenic reactions from SbVP in parenteral products (11) has led to further study in this area in the past few years. Of particular interest is the size range between 2 and 10 μm; however, the measurement of SbVP in this range is a technically challenging and evolving area for both regulators and industry (11,12). Normally for routine quality-control testing of biologic products, the light obscuration (LO) technique is applied, but this is thought to be non-ideal in the 2- to 10-μm range for biological products with translucent protein SbVPs (13,14). A newer technique for measurement of SbVP is flow-imaging microscopy (FIM), which has the capability to classify particulate matter as well as provide size distributions and particulate counts (15,16). Using FIM, algorithms can be produced in order to classify particles based on image characteristics such as shape or translucency (17,18).

Recently, the ErA manufacturing process has been subject to a number of process validation and process robustness studies (19,20) to meet regulatory requirements as well as in order to increase process understanding. As a process understanding measure, SbVP in ErA DP manufacturing and stability settings are routinely analysed using the LO technique. As part of our on-going process understanding efforts around the manufacturing process for ErA, SbVP have also been measured using the FIM technique. In this paper, we compared LO data to FIM measurements for ErA DP. In addition, we compared the SbVP data to the clinical experience for a subset of DP lots.

## MATERIALS AND METHODS

Reagents used were obtained from Sigma (Gillingham, Dorset, UK) unless otherwise indicated. Lyophilised DP vials of *E. chrysanthemi* l-asparaginase (Erwinase®, Porton Down, UK) were obtained from full-scale manufacturing stocks (Public Health England, Porton Down, UK).

### Light Obscuration

The LO measurements were conducted using a Particle Measuring Systems (Boulder, CO, USA) APSS-200 instrument at Reading Scientific Services Limited (RSSL; Reading, UK) according to the US Pharmacopeia (USP) monograph number 788, adapted for 2- to 0-μm size range assessments (21). DP samples were reconstituted in 1–2 mL/vial using 0.9% sterile saline per the clinical product leaflet instructions for ErA. According to the approved and licensed release method used for ErA DP, reconstituted samples were pooled (approximately 20 vials) in order to provide enough sample for LO analysis.

### Flow-Imaging Microscopy

A Fluid Imaging Technologies FlowCAM VS-I (Yarmouth, Maine, USA) with an FC80-7FV flow cell (80 μm depth of field, 700 μm width), and ×10 objective, was used for FIM analyses. The Visual Spreadsheet analysis software was used for data processing, and the software settings are provided in Table I. The vials were reconstituted in 1.5-mL 0.9% sterile saline (Oxoid Limited, Basingstoke, UK) with gentle swirling agitation and no stopper contact, per the clinical product leaflet instructions for ErA. Samples were reconstituted immediately prior to FIM analysis, unless otherwise indicated in the text. For each analysis, the FlowCAM was primed with the sample and the analyses were conducted on 250-μL throughput volumes. Analyses of blank vials (protein and excipient free) were conducted as for the DP analysis; 1.5-mL 0.9% sterile saline was added to a glass vial and used in the FIM analysis. Each analysis took approximately 5 min to conduct. Particle size data were calculated using equivalent spherical diameter (ESD), which is calculated using the mean of 36 independent measurements of Feret diameter.

**Table I:** Software Settings for FIM Analyses

Parameter	Value(s)
Particle segmentation	
Dark threshold	22.00
Light threshold	17.00
Distance to nearest neighbour	7 μm
Close holes	5 iterations
Basic size filter	
Diameter (ESD) min	2.00 μm
Diameter (ESD) max	10,000 μm
Advanced filter	None
AutoImage frame rate	20 frames/s
Flash duration	16.00 μs
Camera gain	0
Analysis volume	250 μL
Run time	Approximately 6 min

### Size-Exclusion Chromatography

The size-exclusion chromatography (SEC) analyses were conducted using a TSKgel G3000SWXL column (Tosoh Bioscience GmbH, Stuttgart, Germany) and a Waters HPLC workstation (Elstree, UK). The running buffer was 100 mM sodium phosphate at pH 7.2 with 100 mM NaCl, and the column eluate was monitored using a variable-wavelength UV detector at 220 nm.

### L-Asparaginase Activity

The l-asparaginase activity assay was conducted using a method based on the Berthelot reaction and methods described in the literature (22,23).

## RESULTS AND DISCUSSION

**Fig. 1:**
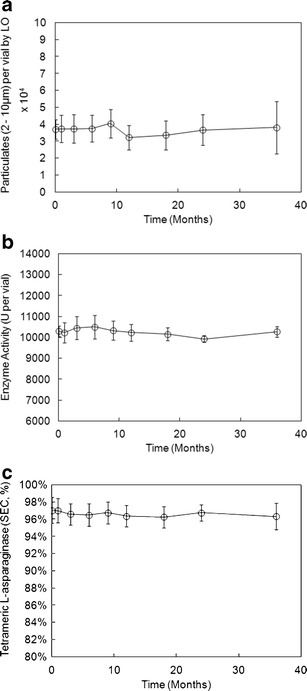
Stability data for ErA, including SbVP content by LO (**a**), enzyme activity (**b**) and aggregation state by SEC (**c**). Each data point represents the mean of measurements of four DP lots, and the *error bars* represent ±1 SD

ErA is presented as a lyophilized dosage form with a high degree of stability under the recommended storage conditions of 2–8°C. Throughout the manufacturing process and prior to aseptic lyophilisation (19), the product is filtered several times using 0.22-μm sterilising-grade or sterile filters, in order to comply with the stringent quality requirements necessary for parenteral pharmaceutical products. The DP is stored as a lyophilized solid and reconstituted with saline solution prior to administration (or analysis in the case of these studies). Data for enzyme activity, 2–10 μm SbVP content as measured by the LO method and soluble aggregates as measured by SEC (Fig. 1) demonstrate the robust stability of the product over the 36-month shelf life. In particular, one notes that protein aggregate content, for both soluble aggregates (SEC) and insoluble aggregates (LO), does not appreciably change or increase over the shelf life of the product. Furthermore, an assessment of soluble aggregates *versus* insoluble aggregates (SEC and LO data) was made, but there was no correlation between the two data sets (data not shown).

**Fig. 2:**
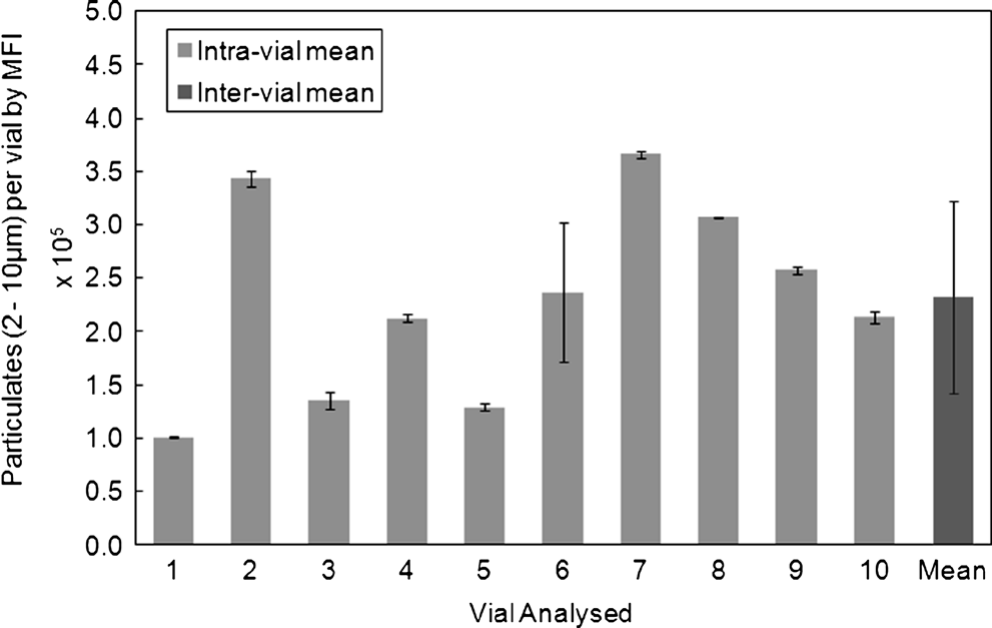
Reproducibility of flow-imaging microscopy analysis of ErA DP. The data (counts of 2–10 μm particles/vial) represent the mean values for four analyses of individual reconstituted vials. The intra-vial mean is indicated by each *bar*, with *error bars* representing ±1 SD. The inter-vial mean (over approximately 40 observations of 10 vials) mean is also shown

Prior to engaging in detailed analysis of SbVP using FIM, the method was defined and qualified during an assay development phase. The final software parameters used with the FIM instrument (Table I) were defined and qualified to ensure reproducibility of results. A population of ten vials from one ErA lot was analysed, with each vial being analysed in four repeats, for a total of 40 analyses (Fig. 2). This strategy allowed both the intra- and inter-vial variability to be assessed. The counts in the 2- to 10-μm ESD particle range show the intra-vial variability, based on four 250-μL aliquots analysed from the same reconstituted vial, to be low, with nine out of ten vials having a percentage coefficient of variance (%CV) ≤6% and eight out of ten vials with a %CV of ≤2.5%. The low intra-vial variance suggests that FIM measurements are very reproducible given a consistent analyte. It is important to note that at this early stage, the %CV as a parameter may not have meaning in absolute terms, therefore it is only provided as a measure of variance relative to the other data in this set. The inter-vial %CV in this data set was much higher at 38.8%, over ten observations. One potential reason for the inter-vial variability observed using FIM is the fact that Erwinase DP is presented as a lyophilized product and that reconstitution of the DP prior to analysis may introduce an inherent variability in the particulate counts from vial to vial compared with other commercial liquid protein formulations. Such variability in SbVP content is not detectable using the pharmacopoeial ErA LO method, as tens of vials are required for pooling to generate one data point using this technique.

**Fig. 3:**
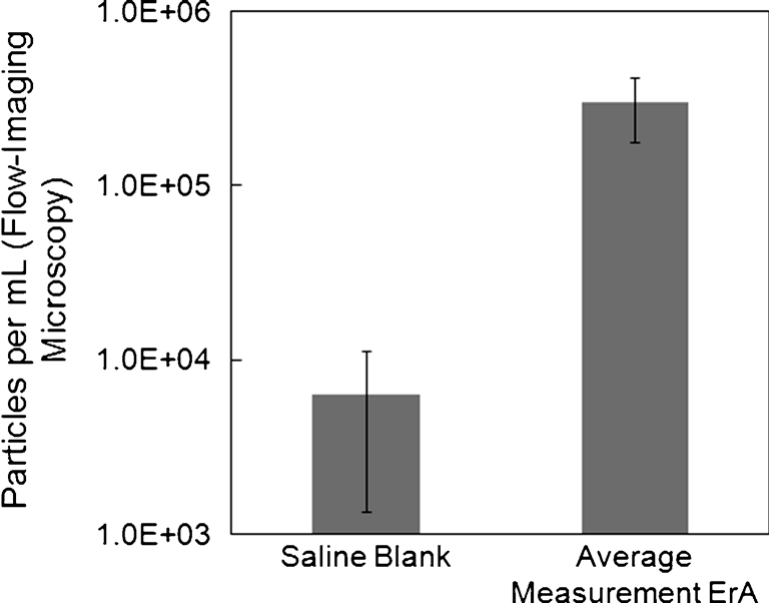
Comparison of SbVP Counts in ErA DP and saline blanks using flow-imaging microscopy. The data (counts of 2–10 μm particles/vial) for the blank represent the mean of 48 individual measurements, and for ErA, the mean of 11 individual lot measurements. The *error bars* represent ±1 SD. The *y*-axis is presented as a logarithmic scale

Theoretically, the vial-to-vial variability in FIM results may be due to presence of air bubbles or other causes introduced during sample preparation. In this study, DP samples were reconstituted according to the approved clinical instructions for Erwinase administration, so as to provide an accurate representation of the material that the patient receives. It is important to note that during this study, special care was taken to conduct the reconstitution procedure in a consistent way. However, the variability in the vial-to-vial FIM measurements is not believed to be due to sample handling or the presence of air bubbles, based on analyses of blank vials consisting of 0.9% saline without the presence of protein. These analyses (Fig. 3) indicate that any matrix-based or sample-handling-based variability is not responsible for the overall variance of ErA FIM measurements, as the background particle counts are nearly two orders of magnitude less than the typical ErA counts. While some background contribution to the particle counts is evident, it is too low to account for the variance observed in the ErA DP vial-to-vial measurements. It is worth noting, however, that these measurements of background particulate counts were made using diluent in the absence of protein. It is possible that other effects due to solution properties (including matrix viscosity or the presence of protein) may have also played a role in microbubble formation and therefore contributed to the vial-to-vial variance.

Interestingly, the contribution to the particulate count background, though likely very low compared with the protein contribution, appeared to be mainly from the 0.9% saline solution, the approved clinical diluent used for ErA DP administration. FIM analyses of 18.2 MΩ water resulted in counts in the region of 100 particles/mL, which is more than one order of magnitude lower than the saline-blank counts. Degassing of the blank solutions was also evaluated but was not found to substantially change the measured levels of background particles in the 0.9% saline diluents (data not shown). Furthermore, incubation of 0.9% saline over 30 min (measured by FIM every 5 min) did not have an effect on the background particulate counts by FIM (data not shown). Compared with water, saline solutions are known to have lower oxygen solubility (24,25), so it is possible that the background particulate counts observed are due to microbubbles formed during natural degassing on opening the diluent vials, and this may help explain why further degassing had no effect. Based on these results, it is reasonable to conclude that the vial-to-vial FIM variability observed is a result of either true intra-lot, vial-to-vial differences in particulate load or a result of subtle differences in the reconstitution procedure.

**Fig. 4:**
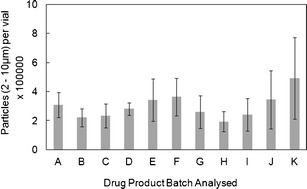
FIM SbVP counts for ErA (particles per vial in the 2–10 μm size range). The results are presented as inter-lot variability for eleven DP lots. Each *bar* in the inter-lot plot represents the mean of four separate reconstituted vials. *Error bars* represent ±1 SD

With these contributions from the matrix background qualified, the inter-lot variability (Fig. 4) was assessed using eleven lots of ErA DP. The DP lots consisted of real-time stability samples held under normal storage conditions (2–8°C), including DP at the start and end of shelf-life (36 months), as well as beyond end of shelf-life (43 months). A number of vials were reconstituted and analysed per batch to provide representative results, and the data obtained show good consistency from batch to batch. Furthermore, the absolute values for SbVP particulate counts are in line with the particulate contents reported for other protein formulations using FIM (26).

**Fig. 5:**
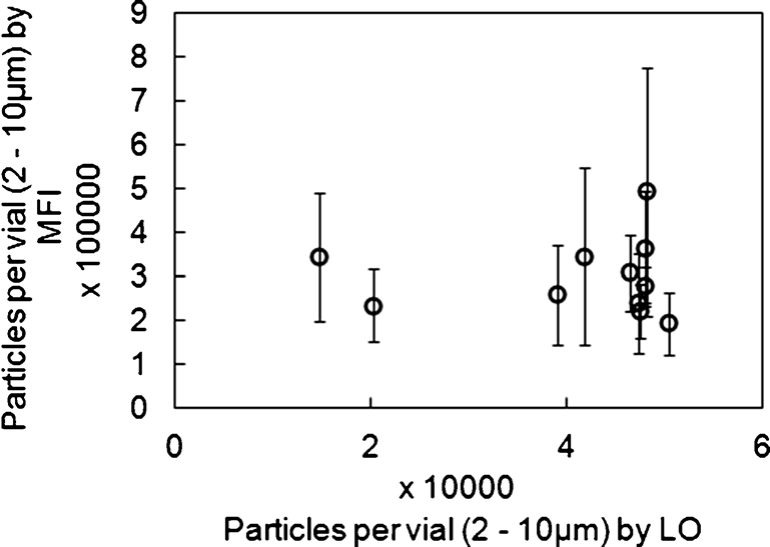
Comparison of SbVP data (particles per vial in 2–10 μm range) for 11 ErA DP lots using both LO and FIM. FIM data represent the mean of four imaged vials. The variability in FIM data (±1 SD *error bars*) are also shown

The FIM data from Fig. 4 were plotted against the corresponding LO data to evaluate whether there was a correlation between the two techniques in the 2- to 10-μm size range (Fig. 5). Each FIM data point in the figure represents the mean value for between 6 and 20 individual vials analysed. As expected based on literature reports (12), the FIM particulate counts are higher than those for the LO technique. Although the FIM 2–10 μm particulate counts cover a range (193,000–493,000 particles/vial), there appears to be no correlation with the LO data (14,698 and 50,356 particles/vial). Ideally, one might expect a linear relationship between the two methods; however, in measurements of ErA DP, the two data sets show little agreement with respect to lot identity. The reasons for this difference are not understood but may be in part due to sample-handling effects as discussed above. One further possibility for the observed variability was a change in the particulate content of reconstituted DP over time. Therefore, a time-course study of ErA DP vials was conducted. The vials were held for varying lengths of time between 5 and 30 min prior to FIM analysis, and the data (data not shown) demonstrated that there was no change in the 2- to 10-μm particulate count over this time period.

One of the potential benefits of analysis by FIM is the possibility of classification of particulate matter using a range of statistical and image processing tools. During each FIM analysis, a large number of images is generated, one image per particle counted. These images may be assessed using computer software and various algorithms to attempt to separate particulate populations from one another. For instance, circular particulates may be more likely to be non-proteinaceous, such as microbubbles, and can be characterised separately from proteinaceous particulate matter (26).

**Fig. 6:**
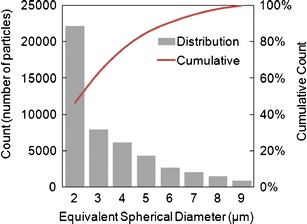
Typical ErA FIM particle size distribution in the size range 2–10 μm. The *histogram bars* show the number of particles in each 1-μm size interval. The curve represents the cumulative percentage of particles less than or equal to the ESD at that point (e.g. at 6 μm approximately 90% of the particles in the 2–10 μm size range are at this size or smaller)

At the most basic level, the FIM technology is able to subclassify particulate populations based on size, down to 1-μm intervals or smaller. Applying this principle to the ErA data discussed above, it is clear that the vast majority of particulate matter is in the lower part of the size range between 2 and 10 μm, as shown in Fig. 6 for a typical ErA DP analysis. In the example analysis shown in the figure, approximately 90% of the particles in the 2- to 10-μm size range are below 6 μm ESD, and approximately 75% are at or below 4 μm ESD.

The typical particle size distribution for Erwinase DP FIM analyses is an important factor to consider when applying further classification filters to the data set. In our experience, although FIM is very good at processing large quantities of particulate images, the image processing software cannot accurately classify particulate images below 4 or 5 μm and can only partially classify particles between 5 and 10 μm. The limitation is related to the resolution of the FIM microscope and camera, and the degree of pixellation involved in images of very small particles. When the particle is very small, the system cannot assign enough pixels to the image to physically allow the computer to distinguish between a circle and other shapes. Furthermore, small particles (2–10 μm) may be slightly out-of-focus due to the depth of field of the flow cell (80 μm), unless they are close to the focal point. This is a difficult technical problem with the currently available FIM instruments. A smaller flow cell might address this problem but could be liable to blockages from larger particulates (ca. >40 μm). Such a flow cell was not available at the time this work was conducted.

**Fig. 7:**
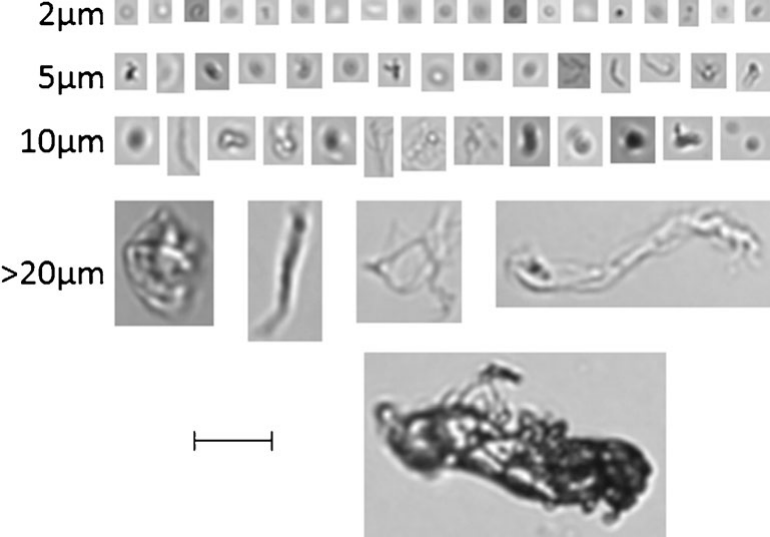
Images of typical ErA SbVP generated by FIM. The ESD as determined by the FIM software is indicated next to each set of images. The *bar* represents 20 μm

Therefore, further classification of ErA FIM data is not straightforward. In a typical ErA DP analysis, approximately 75% of the total particulates in the size range of interest (2–10 μm) are outside the capabilities of the software to adequately determine shape, and the remaining 25% of the population are extremely challenging to classify (Fig. 7). The first particle (top left) in the figure is highly likely to be non-protein in nature but cannot be further defined. A comparison of this image with the images of 2 μm particulates as well as some of the larger particulate images shows that even those particles which have a high degree of circularity and appear quite like the first image cannot be definitively classified as non-proteinaceous in nature.

Despite these challenges, attempts were made to classify 2- to 10-μm particulates in reconstituted ErA DP vials using FIM statistical filters. To illustrate, a single vial of ErA DP was analysed and first a filter was applied to identify the images which were non-protein in nature. Statistical filters for the circularity (0.9–1.0) and compactness (1.0–1.4) were applied, with the values based on standard libraries of images of air bubbles and round SbVP matter. The filters were only able to identify 9% of the images in the 2- to 10-μm size range using these two filters (4,146 particle images out of 47,931 total). A quick review of the images in the remaining 91% (and therefore not identified as circular) identified a substantial number of images which appeared to be non-proteinaceous. Further statistical filters to classify these remaining particles were applied, including those for aspect ratio and intensity as used by other researchers (16), but these did not provide an absolute differentiation of images either, with clear examples of missed non-protein and/or protein particles in the wrong classification sets (data not shown). Fine-tuning the analysis to different aspect ratio or intensity ranges did not yield substantially different results.

However, from this particle classification exercise, a few points become apparent. Firstly, the size analysis based on ESD works quite well and this is apparent when comparing the overall sizes of the particles in Fig. 7. Secondly, the first filter, designed from statistical libraries to find round particles, identified a population of these species but missed a high degree of particles also likely non-proteinaceous. A large proportion of the missed particles were in the smaller end of the size range, and as expected, the instrumentation struggled to classify images at the smallest sizes. When compared with the smaller particles, the images in this last 5- to 10-μm size range are better and easier to visually classify in terms of protein and non-protein species, although there are exceptions even at this size. The discrepancy in selection of particles may be due to the background of the microscopic field, the binary overlay applied to each FIM image by the software or other factors not fully understood.

Although obtaining an accurate classification of 2- to 10-μm particulate species in ErA DP has proven to be difficult, it is clear that protein and non-protein particulates are present. The vast majority of the particles lie at the lower end of the subvisible range, where the resolution of the instrument and the software are impaired, making it very difficult if not impossible to obtain an accurate classification. The higher particle range (>5 μm) represents a small fraction of the total subvisible particle count and is also far from straightforward to accurately classify. A more powerful flow microscope, with better resolution in the 2- to 5-μm size range, would be advantageous at solving this technical issue.

The presence of proteinaceous SbVP in ErA DP, although difficult to absolutely quantify, could at least be confirmed using the FIM technology. The presence of these protein SbVP in ErA is consistent with other biopharmaceutical products as has been described at length in the literature (12,26). As can be observed in Fig. 7, the ErA particulate matter which is clearly proteinaceous in nature has aspect ratios significantly different to 1:1, and have long, string-like morphology or clusters of protein fibrils. These morphologies were observed for ErA particulates greater than 5 μm and are particularly apparent for the larger particles in the 10- to 25-μm size range. It is these large, subvisible aggregates of protein that are potentially of concern with regards to undesired immunogenicity of protein products (27). An undesired immunogenic reaction, such as an allergic reaction, to administration of ErA could have an effect on product efficacy and possibly patient safety.

At the time of writing, no prior reports of SbVP data are known for any l-asparaginase product including ErA and EcA with or without any link to hypersensitivity events. However, in order to increase our product and process knowledge, and understand the potential impact of SbVP on immunogenicity of ErA, a retrospective evaluation of reports of patient allergic reactions was conducted with respect to SbVP content by LO and FIM. Allergic reaction to ErA, which includes both anaphylaxis and hypersensitivity, occurs in approximately 17% of patients (28) and only a subset (20–30%) of the patients who have already had a hypersensitivity reaction to EcA (3,29). In addition, hypersensitivity events from ErA may be less severe than those due to EcA (3).

In ALL treatment with l-asparaginase (including EcA and ErA), the mechanism of allergic reaction is not clear. The formation of anti-asparaginase antibodies is a common occurrence during treatment but does not necessarily result in allergic reaction or hypersensitivity. In one study (30), a significant proportion of patients (36%) treated with EcA and ErA developed anti-asparaginase IgG antibodies, but of these, only half of the patients had an allergic reaction during treatment. Allergic reactions also occurred in the larger patient population that did not develop antibodies, but to a lesser extent (18% of patients in the non-antibody group had a reaction *versus* 56% in the antibody group), and development of antibodies did not affect the overall success of the treatment. A separate study (31) reported measurable anti-asparaginase antibody formation in both EcA and ErA treatment with no difference in the incidence of antibody formation between the two preparations, and no allergic reactions were observed in either arm of the study. Anti-ErA antibodies were also measured (32) in a further study, with none of the patients experiencing hypersensitivity events. A common consequence (in up to 30% of patients) of development of anti-asparaginase antibodies is so-called ‘silent inactivation’ (2), in which the antibodies confer a degree of resistance to the drug but do not result in other clinical symptoms such as allergy.

In order to help understand if SbVP were involved in allergic reactions to ErA, the data from a recently completed clinical study were evaluated. The clinical trial was a safety study in which patients were given ErA at a dosing of 25,000 IU/m^2^ administered intramuscularly six times over 2 weeks (Monday, Wednesday and Friday) to replace each single dose of Oncaspar (PEGylated E coli l-asparaginase) remaining on the individual patient’s treatment schedule. This difference in dosing between Oncaspar and ErA is standard when switching between PEGylated and non-PEGylated l-asparaginases. As one dose of Oncaspar was replaced by six doses of ErA, all patients had more than one ErA dose, and dosing continued until the end of the individuals treatment protocol, or until hypersensitivity occurred. The ErA lot received by each patient was recorded using the patient’s case report form (CRF).

Using clinical data from this trial, we selected a lot of ErA DP that had SbVP counts on the upper edge of the historical database by both FIM and LO (the ‘Target Lot’), and evaluated it for examination of allergic reaction occurrence *versus* the wider clinical database for four other lots with lower SbVP counts. It is important to note that some analyses resulted in high SbVP counts by one technique but low counts by the other; therefore the lot selected has high counts by both (48,000 particles/vial by LO and 280,000 particles/vial by FIM) but does not have the highest counts observed for either technique. However, the control group includes lots with some of the lowest counts (14,000 particles/vial and 20,000 particles/vial for two control lots by LO). Note also that the clinical data set in this analysis does not cover all of the lots depicted in Fig. 4. A total of 1,368 patients were enrolled in the clinical study until the point of study termination, and of these, 228 were treated with the target lot.

The rate of allergic reaction reported for patients who received the selected clinical lot (Table II) is approximately the same or lower than that for the wider clinical study. The odds ratio for the test lot *versus* all other lots was 0.58, indicating that the occurrence of allergic reaction was as likely (or less likely) to occur with the relatively increased levels of SbVP in the target lot. In the ErA (Erwinaze®) product prescribing information (28), the overall rate of allergic reaction is reported as 17%, which is higher than (but roughly the same order of magnitude as) the incidence found in this work. Although unconfirmed for Erwinaze® reactions, allergic reactions are usually immune-system mediated. Undesired immunogenicity is a primary concern with regard to levels of SbVP in parenteral products, but these clinical data suggest that for ErA, increasing SbVP levels do not cause increased incidence of immunogenic reactions or hypersensitivity.

**Table II:** Clinical Data Showing Incidence in Allergic Reactions for an ErA Lot (Target Lot) with Comparatively Higher SbVP Content by LO and FIM, Compared with Incidence for All Other ErA Treatment Lots

Lot ID	Allergic reaction	No allergic reaction	Total	Allergic reactions as % of total
Target lot	15	213	228	6.6%
Not target Lot	124	1,016	1,140	10.9%
Total	139	1,229	1,368	

## CONCLUSIONS

Like many, if not all protein biopharmaceuticals (regardless of dosage form), the presence of SbVP can be detected in reconstituted ErA DP in the 2- to 10-μm size range. The particulate counts appear stable over time by both the established LO technique and the newer FIM technique, however the vast majority of the particulate population was less than or equal to 4 μm in size, where FIM could not definitively classify particles as proteinaceous.

Assessment of the potential clinical impact of these protein SbVP, which are widely observed in protein parenteral products, is the main driver for undertaking the kind of work described in this paper. Occurrences of hypersensitivity, a type of allergic immune response, were found to be approximately the same for an ErA lot with relatively high levels of SbVP by both FIM and LO, compared with the overall occurrence in all clinical lots; this result suggests that presence of protein SbVPs in ErA clinical preparations do not play an important role in triggering allergic reactions. However, each protein biopharmaceutical product is different and therefore new protein products must be evaluated on a case-by-case basis, to build up a better picture of the relationship between protein SbVPs and effects in the clinical setting.
